# Social capital, parental education and depression and anxiety among young people in Latin America: a case-control study

**DOI:** 10.1136/bmjgh-2026-024922

**Published:** 2026-11-11

**Authors:** Hannah Gray, Georgie Hudson, Diliniya Stanislaus Sureshkumar, Catherine Fung, Luis Ignacio Brusco, José Miguel Uribe-Restrepo, Carlos Gomez-Restrepo, Francisco Diez-Canseco, Stefan Priebe, Victoria J Bird, James B Kirkbride

**Affiliations:** 1 Division of Psychiatry, UCL, London, UK; 2 Unit for Social and Community Psychiatry, Queen Mary University of London, London, UK; 3 Department of Psychiatry and Mental Health, University of Buenos Aires, Buenos Aires, Argentina; 4 Department of Psychiatry and Mental Health, Pontificia Universidad Javeriana, Bogota, Colombia; 5 Department of Clinical Epidemiology and Biostatistics, Pontificia Universidad Javeriana, Bogota, Colombia; 6 Universidad Científica del Sur, Lima, Peru; 7 Centre for Psychosocial Medicine, University of Hamburg, Hamburg, Germany; 8 Institute of Public Health and Wellbeing, University of Essex, Colchester, UK

**Keywords:** Public Health, Case-control study, Epidemiology, Mental Health & Psychiatry

## Abstract

**Introduction:**

Growing evidence suggests that social capital is protective against common mental disorders, but how this affects adolescents and young adults living in highly deprived contexts outside of the Global North has received less attention. We investigated whether cognitive (ie, trust, values) and structural (ie, networks, support) social capital were associated with symptoms of depression and anxiety in young people growing up in the poorest half of three cities in South America.

**Methods:**

In this case-control study, we recruited young people, 15–16 and 20–24 years old, from the most deprived half of Bogotá (Colombia), Lima (Peru) and Buenos Aires (Argentina), who did and did not meet threshold criteria for depression and/or anxiety. Following multiple imputation for missing data, we used multivariable logistic regression to test whether individual-level cognitive and structural social capital were associated with case-control status, and we examined whether these were modified by parental education as an indicator of socioeconomic status.

**Results:**

Among 2402 young people (59.8% cases, 65.0% female), those who met case criteria were about half as likely to report living in areas with high cognitive social capital compared with controls (OR: 0.46; 95% CI 0.30 to 0.69). We observed no such association between case status and structural social capital. We did not observe effect modification by parental education.

**Conclusion:**

Higher cognitive social capital was associated with reduced odds of depression and/or anxiety symptoms among youth in economically deprived, urban contexts in South America. This may offer an important community-based resource to promote youth mental health.

**WHAT IS ALREADY KNOWN ON THIS TOPIC**

- Social capital has been associated with depression and anxiety in several contexts, particularly in the Global North, but whether this is a protective factor for youth mental health in highly urbanised deprived contexts in the Global South is unclear. Given rapid urbanisation in South America, we examined this association in the poorest half of three major cities in Argentina, Colombia and Peru.

**WHAT THIS STUDY ADDS**

- In a sample of over 2400 young people living in resource-poor urban contexts in South America, we found that youth who met criteria for depression and/or anxiety were less likely to report high levels of cognitive social capital. Cognitive social capital is one dimension of social capital that measures perceptions of trust, reciprocity and shared norms and values within a community.

**HOW THIS STUDY MIGHT AFFECT RESEARCH, PRACTICE OR POLICY**

- Young people living in highly economically deprived and urbanised parts of three major South American cities reported very low levels of cognitive social capital overall, which was associated with threshold criteria for depression and anxiety. In resource-poor environments, health promotion strategies that foster trust, reciprocity, social connection and community support may represent promising avenues for supporting youth mental health, warranting further investigation in longitudinal studies.

## Introduction

 Depression and anxiety are the most commonly experienced mental disorders worldwide, affecting an estimated 332 million and 359 million people, respectively.^1^ Onset peaks during adolescence and early adulthood, with 73% of anxiety disorders and 37% of depressive disorders estimated to emerge by age 25.^2^ The two frequently co-occur and share many biopsychosocial risk factors.^3–5^ The consequences of these mental health problems in young people extend into later life, predicting poorer educational, employment and relationship outcomes, as well as recurrence of the same or new health issues.^6^ Finding ways to promote mental well-being during adolescence and young adulthood has resultantly been described as both an economic requirement and a moral imperative.^7^

Social capital has attracted interest as a potential resource to be leveraged in this context. Putnam defined social capital as the features of social organisations, such as networks, norms and trust, that facilitate cooperation for mutual benefit.^8^ Higher levels of social capital can enhance well-being by providing emotional, informational and practical support, while fostering a sense of belonging, relatedness and self-esteem.^9 10^ A growing body of literature has demonstrated positive impacts of social capital on health—particularly mental health^11^—across both adult and child populations.^12–15^ Preliminary research also suggests that social capital-based interventions may have potential to protect against and alleviate symptoms of common mental disorders, although current evidence is limited.^16^

Social capital is also a multidimensional construct, and different domains may have differing effects on mental health. For example, some scholars have distinguished between dimensions of cognitive social capital (CSC) and structural social capital (SSC).^17^ CSC refers to perceptions of trust, reciprocity, norms and values within a community, while SSC pertains to the relationships, networks, memberships, organisations, associations and institutions that connect individuals or groups.^18^ CSC has been consistently associated with better mental health,^13 19^ and it is possible that it may exert a greater protective impact on youth mental health in contexts of socioeconomic deprivation, operating to narrow socioeconomic inequalities.^9 20 21^ SSC, on the other hand, has been less consistently linked to positive mental health outcomes^12 13 15 22^; it appears to have a weaker protective effect, particularly in socioeconomically deprived regions.^13^ There is some indication that SSC may even be linked to poorer mental health in low-income settings, possibly because civic participation can impose a burden under conditions of material constraint.^13 19^

Most research on social capital and mental health has been conducted in the Global North.^22^ In recent reviews, only 9 out of 39^13^ and 7 out of 73^23^ studies of social capital and mental health were situated in low- and middle-income countries (LMICs). Of these 14 studies (accounting for two studies included in both reviews), 12 reported associations between social capital and mental health, suggesting that social capital may be an important yet underexplored determinant of mental well-being in these contexts.

Fewer studies have focused on the role of social capital on youth mental health in Latin America,^24^ one of the most urbanised regions in the world.^25^ Young people in the region face intersecting structural challenges—including poverty, unemployment, violence and socio-political instability—that contribute to poorer mental health outcomes.^26 27^ Lack of access to social resources has been identified as a key driver of mental health disparities linked to economic disadvantage and urban living.^28 29^ It follows that promoting social capital might offer a way to reduce socioeconomic inequalities in youth mental health.^23 30^ Better social capital has been linked to reduced depression, anxiety and stress in general adult samples,^31^ including those exposed to armed conflict in South America,^32 33^ although not in adolescents exposed to such conflict.^34^ In Brazil, social capital has also been associated with internalising and externalising disorders in preschool children in a highly urbanised sample from São Paulo,^35^ and with depressive symptoms in both high school^24^ and university students.^36^

Few studies have directly tested whether the association between social capital and its constituent dimensions on mental health outcomes is modified by socioeconomic status (SES). Those that have done so have reported mixed findings, including some evidence for a stronger positive association between social capital and mental well-being among individuals who are more socioeconomically deprived.^37^ However, this research—as well as the wider literature on health outcomes of social capital—is often limited by use of general measures of subjective well-being, and inconsistent definitions and measures of social capital, which mostly fail to account for its multidimensional nature.^23 37^

In the current study, we investigated whether individual-level CSC and SSC were associated with symptoms of depression and anxiety among a large sample of adolescents and young adults living in the most deprived half of three South American capital cities: Buenos Aires in Argentina, Bogotá in Colombia and Lima in Peru. We also sought to understand whether these associations were modified by parental education as an indicator of SES. We hypothesised that: (1) higher CSC and SSC would be associated with lower odds of threshold criteria for depression and/or anxiety in young people and that (2) these associations would be moderated by parental education, such that the association between CSC and case-control status would be stronger for young people with lower parental education, but that the association between SSC and case-control status would be weaker for young people with lower parental education.

## Methods

### Study design and participants

We conducted a secondary analysis, using data from the OLA case-control study, a three-country study of the risk and protective factors for depression and anxiety among youth living in deprived urban areas—Buenos Aires (Argentina), Bogotá (Colombia) and Lima (Peru)—in South America.^38^ Full methods have been detailed elsewhere,^38 39^ but briefly, we used available routine indicators of socioeconomic development to identify the 50% most deprived districts in each city. In Bogotá and Lima, we used the United Nations Development Programme’s Human Development Index^40^ and in Argentina, we used the Unsatisfied Basic Needs Index.^41^

We identified participants, aged 15–16 and 20–24 years old, via a locally-adapted sampling strategy. Across each site, we primarily identified educational establishments (secondary schools and universities) and community organisations (governmental and non-governmental organisations) that provided services to young people. Institutions that agreed to participate assisted with identifying eligible participants, recruitment and screening, although implementation differed slightly according to local needs and resources within each site.^38^ To reach young people who may not have formally engaged with these institutions, we also used social media advertising (Lima) and snowball sampling (Buenos Aires).

At each site, we aimed to recruit 340 young people who met threshold criteria for anxiety or depression (cases), and a further 340 individuals randomly selected from those who did not meet criteria (controls). This sample size was informed by power calculations outlined in the OLA study protocol.^39^

To be included, participants were required to be aged between 15–16 or 20–24 years, and to have capacity to provide informed consent (or assent with parental consent for those under 18 years). Participants were excluded on the basis of any severe mental illness (psychosis, bipolar disorder or schizophrenia), diagnosed learning disability or insufficient fluency in the Spanish language to be able to complete the survey.

### Measures

Measures were all previously validated in the Spanish language and for use by young people in Latin America. Survey data were all self-reported.

### Outcome

Case-control status for depression and/or anxiety was the outcome of the present study. Depression was measured using the Patient Health Questionnaire-8 (PHQ-8),^42^ and anxiety using the Generalised Anxiety Disorder-7 (GAD-7).^43^ Cases were defined by established cut-off scores of 10 or more on the PHQ-8 and/or GAD-7, indicating moderate to severe symptoms. Controls were defined by scores of less than 10 on both scales. We have previously shown that both measures have robust psychometric properties in this sample,^44^ and other studies in Latin America have also confirmed their reliability and validity.^45 46^

### Exposures

Dimensions of social capital were measured at the individual level using the Short Social Capital Assessment Tool (SASCAT).^47^ The SASCAT was developed for use in LMICs and has been recommended over other measures for assessing social capital in these settings.^22^ Its validity and reliability have been demonstrated across contexts,^48^ and the Spanish version has been validated for use in Latin American countries, including Peru.^47^ The questionnaire was designed to measure cognitive and structural dimensions of social capital, allowing for independent analysis of these components. All SASCAT items refer to relations within the community, as defined by local administrative boundaries.

CSC was assessed based on four items (trust, social harmony, perceived fairness and sense of belonging), while the SSC was assessed from five items (group membership; support (emotional, economic or other) received from individuals and groups; and involvement in citizenship activities (talking to local authorities or joining with neighbours to solve a problem)), all within the previous year. For the present study, several items were adapted to include groups that were more relevant to adolescents and young people (replacing ‘credit/funeral’ and ‘other’ with ‘food support’, ‘youth’, ‘arts’ and ‘school or university committee’ groups). This is consistent with author recommendations that the SASCAT be adapted to improve its relevance to the local context^19^ and aligns with previous applications of the measure, including an adapted version used with Colombian adolescents.^34^ Following the approach of the SASCAT authors, we reverse coded the negatively worded ‘perceived fairness’ CSC item, so that higher scores indicated higher social capital.^47^ In line with guidance,^19^ we summed yes/no responses on CSC items to derive a total score and categorised this into ‘low’ (0–2) and ‘high’ (3–4) CSC. For the SSC subdomain, we again followed the approach of the SASCAT authors to rate each item as 0 (no involvement), 1 (one form of involvement) and 2 (two+ forms of involvement). In the absence of formal guidance on deriving a total score, we summed these SSC scores and categorised them into ‘low’ (0–1), ‘medium’ (2–3) and ‘high’ (4–6) SSC.

We also retained a continuous version of each social capital variable (with CSC scores ranging from 0 to 4 and SSC scores from 0 to 6) for the purpose of post hoc sensitivity analyses.

### Effect modifier

Respondents reported the highest level of their parents’ education, which we used as an indicator of SES; this provided a standard measure across the three countries. Parental education was obtained by direct interview with parents for adolescents in Bogotá or otherwise via self-report by the participant. Due to the small number of respondents reporting the lowest category of parental education (‘no formal education’), we made the post hoc decision to combine this category with the next category (‘some primary education’). Responses were therefore categorised into three groups: ‘no formal or some primary education’, ‘some secondary education’ and ‘some higher education’, and we took the highest level of education of either parent.

### Confounders

We selected several variables, a priori, as potential confounders based on previous research and constructed a directed acyclic graph to model the hypothesised confounding structure between social capital domains and case-control status (see online supplemental figures S1 and S2): age, gender identity, parental psychopathology, substance use and life events.^49–55^ Following peer review, setting was additionally included as a potential confounder in the adjusted models.

We categorised age into two groups: 15–16 years and 20–24 years. Gender identity was categorised as male, female or other. Parental psychopathology was assessed by asking participants whether their mother and/or father had ever been treated for a mental health problem (categorised as neither, one or both). Lifetime substance use was assessed using items from the Alcohol, Smoking and Substance Involvement Screening Test adapted for use with adolescents.^56^ Respondents were asked whether they had ever used tobacco, alcoholic beverages, marijuana, cocaine, amphetamines/other stimulants, inhalants, tranquillisers/sleeping pills, hallucinogens or opiates. The number of substances used was summed to give a score from 0 to 9. We used an adolescent-appropriate version of the Life Events Checklist^57^ to obtain participant-reported experiences of major life events during their lifetime. 30 life events were included on the checklist, including a relative or close other having had a serious illness or injury, having changed schools and having been the victim of an assault or robbery. We treated this variable as a continuous variable with a possible range of 0–30. Setting was categorised as Bogotá, Lima or Buenos Aires.

### Statistical analyses

#### Missing data

We treated ‘I don’t know’ responses as a missing data problem and used multiple imputation by chained equations to impute all missing data for exposure, covariate and moderator variables. This approach is appropriate when it is plausible to assume that data are missing at random (MAR).^58^ Auxiliary variables were included in the imputation model to provide information about missing values and enhance the plausibility of the MAR assumption. The final set of auxiliary variables was: four items from the Life Events Checklist^57^ with complete data for all participants, a binary indicator of smartphone ownership, an ordinal measure of current education level ranging from ‘no formal education’ to ‘completed university education’, a categorical measure of employment status and age in years. 20 imputed datasets were generated using appropriate regression models within the chained equations framework (logistic, ordinal logistic or multinomial logistic regression, depending on the variable type), and estimates were pooled across datasets. For social capital variables and major life events, missing data were imputed at the item level and then aggregated using passive imputation to create total scores. Three participants with missing gender data were dropped to allow the multiple imputation model to converge and were excluded from further analyses.

Primary analyses were conducted using our imputed dataset, which were combined according to Rubin’s rules. For comparison, analyses were repeated excluding participants who were missing data on any variables of interest to run complete case analyses.

#### Analysis

We first estimated the proportion of missing data on exposure variables, covariates and effect modifiers (no missing data were present on the outcome) and compared differences between the complete case sample and those with some missing data using appropriate descriptive statistics, including χ^2^ tests (for categorical variables) and Mann-Whitney U tests (MWU; for continuous variables that were not normally distributed, as indicated by the Shapiro-Wilk test). Next, we reported descriptive statistics by case-control status for those with complete data on each covariate of interest, as well as the Pearson correlation between CSC and SSC continuous scores.

For our primary analyses, we fitted unadjusted and fully adjusted logistic regression models to estimate whether higher levels of SSC and/or CSC were associated with lower odds of depression/anxiety caseness. To examine possible effect modification between each social capital domain and parental education on case-control status, we compared our fully-adjusted model with two sequential models fitted with an interaction term between CSC and SES and SSC and SES, respectively. ORs and 95% CIs were reported.

Due to the small number of observations for low CSC, we conducted a post hoc sensitivity analysis using continuous versions of our social capital variables to compare the results with those from our main analysis, which used categorical variables (see online supplemental table S2). We also conducted post hoc power calculations to estimate the power to detect an interaction effect between CSC and parental education, as both categorical and continuous constructs, given our sample size and distribution across these parameters at observed values (see online supplemental table S5). All analyses were conducted using Stata V.19.0.^59^

### Patient and public involvement

The OLA study partnered with several youth arts-based organisations in our three settings, through which we established a panel of young ambassadors who participated in the co-production of a range of arts-based activities to disseminate our findings to local community groups. Further information can be found on the study website: https://theolastudy.com.

## Results

### Sample characteristics and missing data

A total of 1437 cases (59.8%) and 965 controls (40.2%) were recruited into the OLA study, giving a sample size of 2402, following the identification, screening and confirmed eligibility of 6176, 4973 and 3849 initial participants, respectively (see Gomez-Restrepo *et al*^38^ for full details). Complete data were available for 397 (16.5%) of respondents: 223 cases (15.5% of cases) and 174 controls (18.0% of controls). This high level of missingness was primarily due to missing responses to individual CSC items on the SASCAT: Because the CSC score was coded as missing if any of its four component items were missing, the proportion of respondents missing a total CSC score (77.4%) was substantially higher than the overall proportion of missing responses across CSC items (34.0%). Excluding CSC scores, complete data were available for 1735 (72.2%) of respondents: 1014 cases (70.6%) and 721 controls (74.7%). Respondents who were missing data were more likely to be from Colombia or Peru (p=0.04), 15–16 years old (p=0.01) and to have parents with lower or secondary levels of education (p=0.05) (see online supplemental table S1 for sample characteristics by complete data status).

In our total case sample (n=1437), 454 (31.6%) met case criteria based on exceeding the threshold for depressive symptoms only, 163 (11.3%) had anxiety symptoms only and 820 (57.1%) met threshold criteria for both. A greater proportion of cases compared with controls were female (71.5% vs 55.2%), had parents with higher education (43.2% vs 34.7%) and had parent(s) who had received mental health treatment (13.9% vs 9.5%). About 55% of each group belonged to the 20–24 year old group. Cases reported higher levels of lifetime substance use and higher exposure to adverse life events (all p<0.001, table 1). Cases were more likely to report low CSC than controls (p<0.001), but we found no difference in SSC levels between these groups (p=0.18). There was no evidence of a correlation between cognitive and structural dimensions of social capital (r (532)=−0.02, p=0.58).

**Table 1:** Sample characteristics by case-control status

Variable	Total sample, N=2402	Controls, n=965 (40.2%)	Cases, n=1437 (59.8%)	Comparison test, χ^2^ (df)*; p value/MWU†; p value
City				
Buenos Aires	621 (25.9%)	280 (29.0%)	341 (23.7%)	χ^2^ (2): 15.4, p<0.001
Bogotá	965 (40.2%)	344 (35.6%)	621 (43.2%)	
Lima	816 (34.0%)	341 (35.3%)	475 (33.1%)	
Age group				
15–16	1080 (45.0%)	435 (45.1%)	645 (44.9%)	χ^2^ (1): 0.009, p=0.93
20–24	1322 (55.0%)	530 (54.9%)	792 (55.1%)	
Age (15–16)				
Median (IQR)	15 (15–16)	15 (15–16)	15 (15–16)	MWU: 0.4, p=0.71
Age (20–24)				
Median (IQR)	21 (20–23)	21 (20–23)	22 (20–23)	MWU: −0.6, p=0.57
Gender				
Male	815 (33.9%)	428 (44.4%)	387 (26.9%)	χ^2^ (2): 80.6, p<0.001
Female	1560 (65.0%)	533 (55.2%)	1027 (71.5%)	
Other	24 (1.0%)	4 (0.4%)	20 (1.4%)	
Missing	3 (0.1%)	–	3 (0.2%)	
Structural social capital				
Low	474 (19.7%)	197 (20.4%)	277 (19.3%)	χ^2^ (2): 3.4, p=0.18
Medium	1333 (55.5%)	513 (53.2%)	820 (57.1%)	
High	541 (22.5%)	231 (23.9%)	310 (21.6%)	
Missing	54 (2.3%)	24 (2.5%)	30 (2.1%)	
Cognitive social capital				
Low	377 (15.7%)	135 (14.0%)	242 (16.8%)	χ^2^ (1): 28.74, p<0.001
High	165 (6.9%)	100 (10.4%)	65 (4.5%)	
Missing	1860 (77.4%)	730 (75.6%)	1130 (78.6%)	
Parental education				
None/some primary	339 (14.1%)	151 (15.6%)	188 (13.1%)	χ^2^ (2): 16.9, p<0.001
Some secondary	1009 (42.0%)	436 (45.2%)	573 (39.9%)	
Some higher	955 (39.8%)	335 (34.7%)	620 (43.1%)	
Missing	99 (4.1%)	43 (4.5%)	56 (3.9%)	
Substance use				
Median (IQR)	1 (0–2)	1 (0–2)	1 (1–3)	MWU: −7.5, p<0.001
Adverse life events				
Median (IQR)	9 (6–13)	8 (5–11)	10 (7–13)	MWU: −10.7, p<0.001
Parental mental health treatment				
Neither	1532 (63.8%)	667 (69.1%)	865 (60.2%)	χ^2^ (2): 17.1, p<0.001
One parent	256 (10.7%)	85 (8.8%)	171 (11.9%)	
Both parents	36 (1.5%)	7 (0.7%)	29 (2.0%)	
Missing	578 (24.1%)	206 (21.3%)	372 (25.9%)	

* χ2: Pearson’s χ2 test used to test for differences in the distribution of categorical variables between cases and controls.

† MWU: MWU test used to test for differences in the median value of continuous variables between cases and controls.

df, degrees of freedom; MWU, Mann-Whitney U.

### Associations between social capital domains, SES and depression and anxiety

In our univariable model, participants who met case thresholds for depression and/or anxiety were less likely to report exposure to high CSC (OR: 0.38; 95% CI 0.30 to 0.69; table 2, figure 1), which did not substantially attenuate after adjustment for a priori confounders, including SSC and parental education (OR: 0.46; 95% CI 0.30 to 0.69). Across our univariable and multivariable models, we found no evidence of an association between case status and SSC, either for high vs low SSC or medium vs low SSC (table 2, figure 1). Unexpectedly, we observed that participants whose parents had completed some level of higher education were more likely to be cases than controls, compared with participants whose parents’ highest level of education was either ‘no formal or some primary’ (adjusted OR: 0.73; 95% CI 0.55 to 0.98) or ‘some secondary’ (adjusted OR: 0.79; 95% CI 0.64 to 0.98). Results obtained using our imputed dataset were very similar to complete case analysis results (online supplemental table S3), supporting the plausibility of the MAR assumption in this dataset. We found no evidence that parental education modified observed associations between CSC or SSC and odds of depression and/or anxiety (all p>0.05, see online supplemental table S4), but our estimated power was very low (online supplemental table S5), at just 8.5% to detect an interaction OR of 0.86 (as observed for the parameter between CSC (low vs high) and parental education (primary vs higher) at our given sample size and exposure distribution levels. Our findings were consistent in post hoc sensitivity analyses (online supplemental table S2), where we substituted categorical measures of CSC and SSC for continuous scores; these analyses were sufficiently powered to detect interactions at least as large as those observed (adjusted OR: 0.90; power: 82.1%; online supplemental table S6) between social capital and parental education on case-control status, should they have existed, but consistent with our main analyses none were observed (online supplemental table S2).

**Figure 1:**
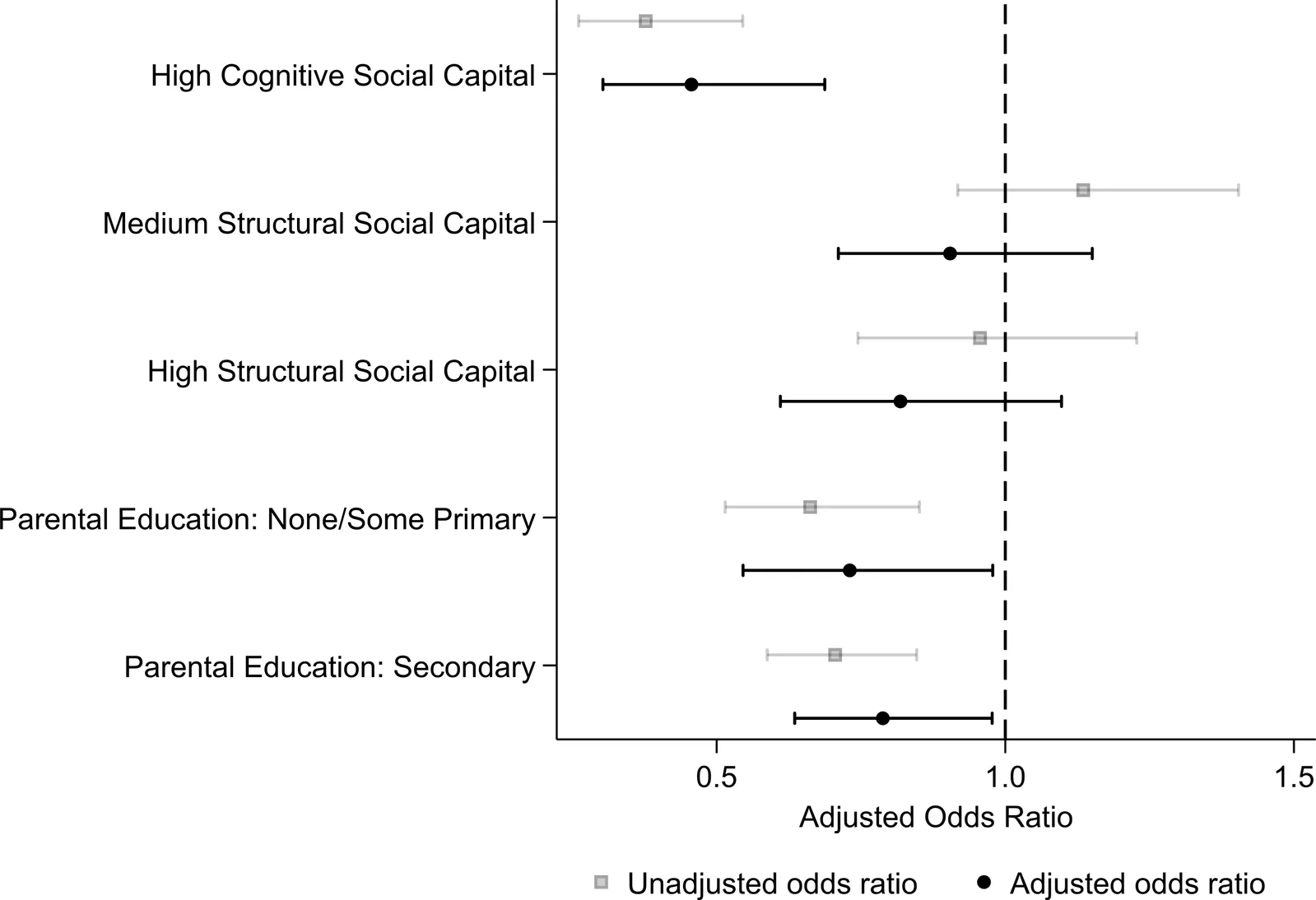
Coefficient plot from logistic regression models. Note: odds of depression/anxiety associated with CSC (reference category: low), SSC (reference category: low) and parental education (reference category: higher education), across unadjusted models and models adjusted for confounders, using imputed dataset. CSC, cognitive social capital; SSC, structural social capital.

**Table 2:** Unadjusted and adjusted results of logistic regression predicting youth depression/anxiety using imputed dataset

	Unadjusted		Adjusted	
	OR	95% CI	OR	95% CI
Structural social capital (ref. low)				
Medium	1.14	0.92 to 1.40	0.90	0.71 to 1.15
High	0.96	0.74 to 1.23	0.82	0.61 to 1.10
Cognitive social capital (ref. low)	**0.38**	**0.26 to 0.55**	**0.46**	**0.30 to 0.69**
Parental education (ref. higher education)				
No formal/some primary education	**0.66**	**0.51 to 0.85**	**0.73**	**0.55 to 0.98**
Some secondary education	**0.71**	**0.59 to 0.85**	**0.79**	**0.64 to 0.98**
City (ref. Bogotá)				
Buenos Aires	**0.68**	**0.55 to 0.83**	**0.74**	**0.58 to 0.95**
Lima	**0.77**	**0.64 to 0.94**	**0.63**	**0.51 to 0.80**
Age group (ref. 15–16 years)	1.01	0.86 to 1.19	**0.78**	**0.64 to 0.96**
Gender (ref. male)				
Female	**2.13**	**1.79 to 2.53**	**2.33**	**1.91 to 2.83**
Other	**5.53**	**1.87 to 16.32**	**4.71**	**1.51 to 14.66**
Substance use	**1.20**	**1.13 to 1.27**	**1.16**	**1.08 to 1.24**
Adverse life events	**1.11**	**1.09 to 1.14**	**1.09**	**1.06 to 1.12**
Parental mental health treatment (ref. neither parent)				
One parent	**1.49**	**1.12 to 1.97**	1.25	0.92 to 1.70
Both parents	**2.76**	**1.21 to 6.28**	**2.80**	**1.15 to 6.82**

Statistically significant effects are shown in bold.

Regression analyses were conducted using the imputed dataset, comprising a sample of 2399 observations.

## Discussion

### Main findings

We found strong evidence that higher CSC was associated with reduced likelihood of meeting threshold criteria for depression and/or anxiety in this sample of young people living in highly deprived, urbanised parts of three major cities in South America, in line with our first hypothesis. Young people with high CSC had about half the odds of experiencing clinically significant symptoms compared with those who reported low CSC; these results were robust to adjustment for several important confounders, including parental psychopathy, substance use, parental education and adverse life events. Contrary to our hypotheses, we did not observe any association between SSC and case status for depression and/or anxiety.

### Comparison with previous research

The strong inverse association we observed between high CSC and lower odds of case status for depression and/or anxiety among young people in this sample aligns with previous research on this dimension of social capital and mental health outcomes,^13^ including those from Latin America.^36^ Importantly, almost 70% of young people in our study reported low CSC, characterised by a perceived lack of trust, social harmony, fairness and sense of belonging within their communities. Similar patterns have been observed in other studies of Latin American youth.^34^ Although causality cannot be inferred from case-control study designs, our work suggests further longitudinal research embedded in stronger causal frameworks is warranted to elucidate whether primary prevention strategies that deploy interventions intended to improve young people’s access to CSC would be effective in reducing the high burden of youth mental ill-health documented in Latin America and beyond.^60–62^ Not all studies have found that social capital protects against youth mental health in Latin America.^34^ Future research needs to ensure levels of support at multiple levels—including family, school and community—are simultaneously measured to identify the level of intervention at which promoting social support or cohesion may be most effective.

An earlier report from the OLA study aimed to explore a broad range of putative factors associated with case-control status, and also reported no association between SSC and symptoms of depression and/or anxiety.^38^ That study did not include CSC, and did not investigate confounding structures that may have affected the specific association between social capital and mental health, nor did it consider potential effect modification by SES. We also extended that study by treating ‘I don’t know’ responses as missing data and including a multiple imputation routine to recover missing data, but our findings are consistent with each other and with the previous literature to show that SSC is not strongly associated with depression or anxiety.^13 14^ These findings support the view that cognitive rather than structural elements of social capital are most relevant to youth mental health.

There is growing evidence from other settings that the protective association between social capital and youth mental health and behavioural outcomes is stronger for children from lower SES backgrounds, measured variably in different studies on this topic.^9 20 37^ For example, in samples of Flemish and New Zealand adolescents, De Clercq *et al* and Aminzadeh *et al* found that adolescents who were more socioeconomically deprived benefited from a greater protective effect of social capital on well-being.^9 20^ Three further studies identified in the systematic review by Vyncke *et al*^37^ also identified such trends,^63–65^ although one did not.^66^ We found no evidence of such effect modification in this Latin American sample of young people, particularly in sensitivity analyses with CSC and SSC modelled as continuous variables which were well powered to detect interactions with parental education, should they have existed. To our knowledge, ours is the only study conducted on this topic outside of the Global North. The contexts in which youth in our study areas lived were—by design—very different from those of previous studies conducted on this issue. For example, we purposefully recruited participants from the most deprived half of three major urban centres in Argentina, Colombia and Peru, which may have restricted the modifying role of individual-level SES—as measured by a single measure of parental education in our study—on the relationship between social capital and youth mental health. Unexpectedly, young people whose parents reported some level of higher education were more likely to meet threshold criteria for depression and/or anxiety. Although this contrasts most previous research, the protective effect of parental education on child mental health has been shown to vary by geographical location and may attenuate in low-income contexts.^67^ One possible explanation is that higher levels of parental education increase mental health literacy and, consequently, self-reported mental health symptoms.^68^

### Strengths and limitations

Our multicentre study of the effect of social capital and SES on youth mental health in economically deprived parts of South America used validated and reliable measures in a large sample to understand the potential relationship between these factors. We referred to methods outlined by authors of the SASCAT^47^ when categorising social capital scores, and given the low proportion of our sample who reported ‘high’ levels of CSC, we replicated our results in a post hoc sensitivity analysis using continuous scores. We controlled for important covariates which we selected based on prior research. Missing data were handled using multiple imputation, and the robustness of findings was assessed by comparing results from the imputed datasets with complete case analyses. We included auxiliary variables that predicted both missingness and exposure status in the imputation model to enhance plausibility of the MAR assumption.

Despite these strengths, several limitations warrant important consideration. First, due to the case-control design, we could not establish causality. It is possible that components of CSC, such as perceived trust and social harmony, were influenced by mental health status, and longitudinal studies are required to elucidate this. Second, we cannot exclude the introduction of same-source bias,^69^ given that data on exposure and outcome were provided by the same individuals. Unfortunately, we did not have independent measures of social capital in this study. Despite these concerns, longitudinal evidence has shown that self-reported CSC is a stronger predictor of mental health than mental health is of CSC,^13 14^ and these associations persist when ecological measures of social capital are used in place of individual measures vulnerable to same-source bias.^13^ Third, although our sampling strategy was guided by epidemiological principles that allowed us to recruit participants from the poorest communities in our three settings, methods to recruit young people from these communities varied by local pragmatic choices, and included snowball and convenience sampling methods (see Gomez-Restrepo *et al*^38^ for full details); this may have introduced selection bias into our results, particularly if we were more likely to recruit those who participated in formal education or the social settings where we sought to recruit young people. Fourth, parental education may have provided a limited proxy measure of SES^70^; we did not have other measures of SES available in the OLA dataset that were directly comparable between countries. Fifth, we were underpowered to detect interactions between categorical measures of social capital and parental education, given the distribution of participants across these variables and our observed sample size. Caution is warranted in interpreting the results from these interaction analyses, although well-powered sensitivity analyses that used continuous social capital scores supported this conclusion. Sixth, although we adjusted for substance use and adverse life events in our analyses, it is plausible that these variables acted as mediators in the pathway between social capital and mental health. We considered the potential for overadjustment and included these variables based on our a priori confounding structure informed by careful review of the existing literature.^50 51^ Finally, data used in the present analyses were collected during the COVID-19 pandemic, which could have impacted both the social relations and mental health of Latin American youth,^71^ potentially influencing the associations observed in our study.

### Implications

The present study calls attention to the strong association between CSC and symptoms of depression/anxiety among South American youth living in deprived urban regions, particularly given that the majority of young people in our sample reported low CSC at baseline (over 87%; table 1). These findings underscore the need for further investigation into the causal nature of this relationship. Longitudinal research is warranted to determine whether CSC might represent a modifiable target for improving youth mental health outcomes in Latin American contexts, where access to mental healthcare is often limited.^72^ The lack of a clear association we observed between SSC and mental health outcomes highlights the importance of distinguishing between different dimensions of social capital when seeking targets to achieve meaningful improvements in mental well-being. If social capital is to be considered as a potential target for reducing mental health inequalities among disadvantaged youth, greater attention may be warranted to context-specific approaches that prioritise the quality, rather than simply the quantity, of community connections.^73^ There is some preliminary but inconclusive evidence for the efficacy of such CSC interventions implemented in urban communities in LMICs.^16^ Moreover, in our previous study we found that youth who engaged in different activities had differential mental health outcomes.^38^ For example, controls were more likely to report engagement in sports activities than cases, but less likely to report engagement in arts activities. Although we need to understand the social contexts in which these activities took place (as groups or individually), it is tentatively possible that sports activities here were more likely to be group-based or team-based activities whereas arts were more individualistic pursuits. We now require longitudinal evidence to determine whether activities and communities that facilitate CSC could serve as protective resources for youth mental health.

## Conclusion

Higher CSC, but not SSC, was associated with reduced odds of depression and/or anxiety symptoms among youth in economically deprived urban contexts in South America. These associations were not modified by parental education. This may offer an important community-based resource to promote youth mental health, warranting further investigation through longitudinal research.

## Supplementary material

10.1136/bmjgh-2026-024922 online supplemental file 1

10.1136/bmjgh-2026-024922 online supplemental file 2

## Data Availability

Data may be obtained from a third party and are not publicly available.
