# Supplementary material for: Social capital, parental education and depression and anxiety among young people in Latin America: a case-control study
Source: BMJ Glob Health. 2026 Nov 11;11(11):e024922. doi: 10.1136/bmjgh-2026-024922 (PMC13756383; doi:10.1136/bmjgh-2026-024922)
Supplement: Supplementary file 1 [file bmjgh-11-11-s001.docx]

### BMJ Global Health Author Reflexivity Statement

Adapted from Morton, B., Vercueil, A., Masekela, R., Heinz, E., Reimer, L., Saleh, S., Kalinga, C., Seekles, M., Biccard, B., Chakaya, J., Abimbola, S., Obasi, A. and Oriyo, N. (2022), Consensus statement on measures to promote equitable authorship in the publication of research from international partnerships. Anaesthesia, 77: 264-276. <https://doi.org/10.1111/anae.15597>

| **Study conceptualisation** | |
| --- | --- |
| 1. How does this study address local research and policy priorities? | Most countries in Latin America allocate low levels of resources to mental health care, leaving substantial need in the population, particularly amongst youth living in the most socio-economically deprived parts of major urban centres in the region. This research addresses that gap, by investigating some of the determinants of risk of poor mental health. |
| 2. How were local researchers involved in study design? | In each site (Argentina, Colombia, Peru), one to two Principal Investigators based in the region at academic institutions were invited to lead the project as site PIs, and were involved from the outset as costed partners for the full duration of the study, from conception to dissemination. |
| **Research management** | |
| 3. How has funding been used to support the local research team(s)? | Funding provided salary support for local PIs, their staff and all participant recruitment activities, research and dissemination at each site. |
| **Data acquisition and analysis** | |
| 4. How are research staff who conducted data collection acknowledged? | Acknowledged in the manuscript for data acquisition. |
| 5. How have members of the research partnership been provided with access to study data? | Via data sharing between all partners, centrally managed by the Chief Investigator of the OLA study, Prof Stefan Priebe. A transparent analysis and publication strategy was agreed by all lead investigators as an internal study document to guide this process. |
| 6. How were data used to develop analytical skills within the partnership? | Expertise from researchers and academics who were co-investigators at our Global North institutions (UCL, Queen Mary University London) were offered to local sites. Knowledge exchange was established via our network to provide analytic skills to local researchers working on OLA research projects. |
| **Data interpretation** | |
| 7. How have research partners collaborated in interpreting study data? | Critical feedback on the results and their interpretation. |
| **Drafting and revising for intellectual content** | |
| 8. How were research partners supported to develop writing skills? | Via our publication strategy, see point 5. |
| 9. How will research products be shared to address local needs? | Via a series of dissemination events to local policymakers, service providers and non-governmental organisations during the funded part of the OLA study. See <https://www.theolastudy.com> for more details. |
| **Authorship** | |
| 10. How is the leadership, contribution and ownership of this work by LMIC researchers recognised within the authorship? | Via authorship. |
| 11. How have early career researchers across the partnership been included within the authorship team? | Via opportunities to co-author the work. |
| 12. How has gender balance been addressed within the authorship? | Gender balance in authorship reflects the contributions of members of this network to the paper, and the academic cultures that arise in their local jurisdictions. Within this, we have strong female representation in first and senior authorship positions on this paper. |
| **Training** | |
| 13. How has the project contributed to training of LMIC researchers? | Analytical methods have been shared with LMIC researchers involved in the OLA project via meetings and supervision to allow them to apply similar methods to their OLA research. |
| **Infrastructure** | |
| 14. How has the project contributed to improvements in local infrastructure? | The findings from the OLA study have been disseminated via innovative arts-based projects and grassroots organisations to provide support and recognition for youth mental health. |
| **Governance** | |
| 15. What safeguarding procedures were used to protect local study participants and researchers? | Local ethics approval and informed consent and assent procedures. Compliance with all prevailing local public health and other advisory statements in place at the time of the study. |
