# Supplementary material for: Social capital, parental education and depression and anxiety among young people in Latin America: a case-control study
Source: BMJ Glob Health. 2026 Nov 11;11(11):e024922. doi: 10.1136/bmjgh-2026-024922 (PMC13756383; doi:10.1136/bmjgh-2026-024922)
Supplement: Supplementary file 2 [file bmjgh-11-11-s002.docx]

**Supplementary Materials**

*Post hoc power calculations*

Given the absence of observed interactions between cognitive or structural social capital as categorical variables and parental education (see results), and presence of small cells in some exposure categories (Table 2) we estimated statistical power in a *post hoc* analyses. We estimated the statistical power to detect an interaction between low versus high (ref) exposure to cognitive social capital (binary) and exposure to primary versus higher (ref) parental education (four-level categorical variable) on the multiplicative (odds ratio) scale within our case–control design. Power was computed analytically using the expected cell counts under a logistic regression model, given the observed sample size (1,437 cases and 965 controls), the observed joint distribution of CSC by parental education in controls, and the observed main‑effect odds ratios for cognitive social capital and parental education (Table 2). The interaction contrast of interest compared the CSC‑associated odds ratio in those exposed to primary parental education versus those exposed to higher parental education. For each hypothesised interaction odds ratio (θ = 0.4, 0.50, 0.75, 0.86 (as observed, Table S4), 0.9), we derived the expected case cell probabilities under the logistic model, computed the variance of log(θ) from the corresponding 2×2 tables, and applied a two‑sided Wald test at α = 0.05. We repeated this power calculation for CSC as a continuous score, in sensitivity analyses. This approach provided a valid large‑sample approximation to the power of the interaction term in logistic regression. Results from these power calculations are presented in Tables S5 and S6.

**Figure S1: Directed Acyclic Graph (DAG) Used to Identify Confounders of the Association Between CSC and SES**

*Legend*. Directed Acyclic Graph of hypothesised confounding structure between variables. The green line shows the direct causal path between CSC and depression/ anxiety. Confounders (gender, age, substance use, adverse life events, parental psychopathology) were identified a priori as predictors of both social capital and depression/ anxiety. This DAG was constructed using [www.daggity.net](http://www.daggity.net) to estimate the minimal sufficient adjustment set(s) required to estimate the association between CSC and case-control status.

**Figure S2: Directed Acyclic Graph (DAG) Used to Identify Confounders of the Association Between CSC and SES**

Legend. Directed Acyclic Graph of hypothesised confounding structure between variables. The green line shows the direct causal path between SSC and depression/anxiety. Confounders (gender, age, substance use, adverse life events, parental psychopathology) were identified a priori as predictors of both social capital and depression/ anxiety. This DAG was constructed using [www.daggity.net](http://www.daggity.net) to estimate the minimal sufficient adjustment set(s) required to estimate the association between SSC and case-control status.

| **Table S1: Sample characteristics by complete data status** | | | | |
| --- | --- | --- | --- | --- |
|  | | | | |
| **Variable** |  | **Complete data** *n* = 397 (16.5%) | **Incomplete data** *n* = 2005 (83.5%) | **Comparison test**  χ^2^ (df)^a^; *p*-value/ MWU^b^; *p*-value |
| **Case-control status** | Case | 223 (56.2%) | 1214 (60.6%) | χ^2^ (1) = 2.6, *p* = .10 |
|  | Control | 174 (43.8%) | 791 (39.5%) |  |
| **City** | Buenos Aires | 123 (31.0%) | 498 (24.8%) | χ^2^ (2) = 6.6, *p* = .04 |
|  | Bogotá | 150 (37.8%) | 815 (40.7%) |  |
|  | Lima | 124 (31.2%) | 692 (34.5%) |  |
| **Age group** | 15-16 | 156 (39.3%) | 924 (46.1%) | χ^2^ (1): 6.2, *p* =.01 |
|  | 20-24 | 241 (60.7%) | 1081 (53.9%) |  |
| **Age (15-16s)** | Median (IQR) | 16 (15, 16) | 15 (15, 16) | MWU: -0.31, *p* = .76 |
| **Age (20-24s)** | Median (IQR) | 21 (20, 23) | 21 (20, 23) | MWU: 0.84, *p* = .40 |
| **Gender** | Male | 141 (35.5%) | 674 (33.6%) | χ^2^ (2): 0.74, *p* = .69 |
|  | Female | 253 (63.7%) | 1307 (65.2%) |  |
|  | Other | 3 (0.8%) | 21 (1.1%) |  |
|  | Missing | - | 3 (0.2%) |  |
| **Structural social capital** | Low | 76 (19.1%) | 398 (19.9%) | χ^2^ (1): 4.1, *p* = .12 |
|  | Medium | 214 (53.9%) | 1119 (55.8%) |  |
|  | High | 107 (27.0%) | 434 (21.7%) |  |
|  | Missing | - | 54 (2.7%) |  |
| **Cognitive social capital** | Low | 274 (69.0%) | 103 (5.1%) | χ^2^ (1): 0.2, *p* = .65 |
|  | High | 123 (31.0%) | 42 (2.1%) |  |
|  | Missing | - | 1860 (92.8%) |  |
| **Parental education** | None/some primary | 69 (17.4%) | 270 (13.5%) | χ^2^ (2): 6.1, *p* = .05 |
|  | Some secondary | 184 (46.4%) | 825 (41.2%) |  |
|  | Some higher | 144 (36.3%) | 811 (40.5%) |  |
|  | Missing | - | 99 (4.9%) |  |
| **Substance use** | Median (IQR) | 1 (0, 2) | 1 (0, 2) | MWU: -0.4, *p* = .66 |
| **Adverse life events** | Median (IQR) | 9 (6, 13) | 9 (7, 13) | MWU: -0.1, *p* = .91 |
| **Parental mental health treatment** | Neither | 331 (83.4%) | 1201 (59.9%) | χ^2^ (2): 0.79, *p* = .67 |
|  | One parent | 56 (14.1%) | 200 (10.0%) |  |
|  | Both parents | 10 (2.5%) | 26 (1.3%) |  |
|  | Missing | - | 578 (28.8%) |  |

^a^ χ^2^: Pearson's chi-squared test used to test for differences in the distribution of categorical variables between cases and controls; df = degrees of freedom.

^b^ MWU: Mann–Whitney *U* test used to test for differences in the median value of continuous variables between cases and controls.

| **Table S2: Adjusted results of logistic regression predicting youth depression/anxiety from continuous social capital scores using imputed dataset** | | | |
| --- | --- | --- | --- |
|  |  | **Adjusted** | |
|  | **OR** | | **95% CI** |
| **Structural social capital** | 0.95 | | (0.88, 1.03) |
| **Cognitive social capital** (ref. low) | **0.72** | | **(0.62, 0.85)** |
| **Parental education** (ref. higher education) |  | |  |
| No formal/Some primary education | **0.73** | | **(0.54, 0.99)** |
| Some secondary education | 0.80 | | (0.64, 1.01) |
| **City** (ref. Bogotá) |  | |  |
| Buenos Aires | **0.76** | | **(0.59, 0.98)** |
| Lima | **0.64** | | **(0.51, 0.81)** |
| **Age group** (ref. 15-16 years) | **0.78** | | **(0.63, 0.95)** |
| **Gender** (ref. male) |  | |  |
| Female | **2.29** | | **(1.87, 2.81)** |
| Other | **4.25** | | **(1.32, 13.67)** |
| **Substance use** | **1.16** | | **(1.08, 1.25)** |
| **Adverse life events** | **1.09** | | **(1.06, 1.11)** |
| **Parental mental health treatment** (ref. neither parent) |  | |  |
| One parent | 1.23 | | (0.89, 1.69) |
| Both parents | **2.78** | | **(1.14, 6.76)** |
| **Effect modification between social capital (continuous and parental education)** |  | |  |
| CSC x no formal / some parental education (vs. higher education) | 0.90 | | (0.46, 1.26) |
| CSC x some secondary parental education (vs. higher education) | 0.86 | | (0.56, 1.34) |
| SSC x no formal / some parental education (vs. higher education) | 0.90 | | (0.73, 1.67) |
| SSC x some secondary parental education (vs. higher education) | 0.73 | | (0.46, 1.13) |

OR= Odds ratio. 95% CI = 95% confidence intervals. Statistically significant effects are shown in bold.

| **Table S3:** **Unadjusted and adjusted results of logistic regression predicting youth depression/anxiety using complete case analysis** | | | | | | |
| --- | --- | --- | --- | --- | --- | --- |
|  | | | | | | |
|  | **Unadjusted** | |  |  | **Adjusted** | |
|  | **OR** | **95% CI** | | **OR** | | **95% CI** |
| **Structural social capital** (ref. low) |  |  | |  | |  |
| Medium | 0.98 | (0.58, 1.65) | | 0.62 | | (0.34, 1.12) |
| High | 1.20 | (0.66, 2.18) | | 0.78 | | (0.39, 1.55) |
| **Cognitive social capital** (ref. low) | **0.36** | **(0.23, 0.56)** | | **0.54** | | **(0.33, 0.87)** |
| **Parental education** (ref. higher education) |  |  | |  | |  |
| No formal/Some primary education | 0.66 | (0.37, 1.17) | | 0.68 | | (0.34, 1.37) |
| Some secondary education | 0.76 | (0.48, 1.18) | | 0.84 | | (0.50, 1.41) |
| **City** (ref. Bogotá) |  |  | |  | |  |
| Buenos Aires | 0.68 | (0.41, 1.10) | | 0.63 | | (0.35, 1.13) |
| Lima | **0.48** | **(0.30, 0.78)** | | **0.36** | | **(0.20, 0.63)** |
| **Age group** (ref. 15-16 years) | 0.94 | (0.63, 1.42) | | 0.79 | | (0.48, 1.29) |
| **Gender** (ref. male) |  |  | |  | |  |
| Female | **1.93** | **(1.27, 2.94)** | | **2.46** | | **(1.51, 4.00)** |
| Other | - | - | | - | | - |
| **Substance use** | **1.28** | **(1.13, 1.46)** | | **1.20** | | **(1.03, 1.42)** |
| **Adverse life events** | **1.11** | **(1.06, 1.16)** | | **1.09** | | **(1.03, 1.16)** |
| **Parental mental health treatment** (ref. neither parent) |  |  | |  | |  |
| One parent | **1.84** | **(1.01, 3.35)** | | 1.38 | | (0.71, 2.68) |
| Both parents | 3.48 | (0.73, 16.64) | | 3.39 | | (0.62, 18.64) |
| OR= Odds ratio. 95% CI = 95% confidence intervals. Statistically significant effects are shown in bold. | | | | | | |

|  | **Table S4: Odds ratios and p-values for imputed models investigating effect modification of socioeconomic status on the relationship between cognitive and structural social capital and odds of depression/ anxiety** | | | | |
| --- | --- | --- | --- | --- | --- |
|  | |  | | | |
|  | | | **N** | ***p-value*** | **OR (95% CI)** |
| **Structural social capital x Parental education** | | |  |  |  |
| Low x No/ Some primary education | | | 79 | – | – |
| Low x Some secondary education | | | 214 | – | – |
| Low x Some higher education | | | 156 | – | – |
| Medium x No/ Some primary education | | | 178 | .312 | 0.70 (0.35, 1.40) |
| Medium x Some secondary education | | | 541 | .932 | 1.02 (0.61, 1.73) |
| Medium x Some higher education | | | 560 | – | – |
| High x No/ Some primary education | | | 74 | .362 | 0.68 (0.30, 1.55) |
| High x Some secondary education | | | 227 | .693 | 1.13 (0.62, 2.07) |
| High x Some higher education | | | 220 | – | – |
| **Cognitive social capital x Parental education** | | |  |  |  |
| Low x No/ Some primary education | | | 306 | – | – |
| Low x Some secondary education | | | 871 | – | – |
| Low x Some higher education | | | 840 | – | – |
| High x No/ Some primary education | | | 32 | .743 | 0.86 (0.34, 2.19) |
| High x Some secondary education | | | 132 | .702 | 0.89 (0.50, 1.61) |
| High x Some higher education | | | 112 | – | – |

Low social capital and higher education were used as the reference categories.

N represents the number of participants in each exposure category in the analytic sample.

**Table S5: *Post hoc* statistical power calculation at observed values for CSC (low vs. high) x parental education (none/primary vs. higher)**

| **Interaction odds ratio (θ)**^a^ | **Power (%)** |
| --- | --- |
| 0.40 | 81.7 |
| 0.50 | 62.5 |
| 0.75 | 17.5 |
| 0.86 (as observed) | 8.5 |
| 0.90 | 6.7 |

^a^ Interaction odds ratios less than one indicated a stronger protective effect of CSC on case-control status in people with lower parental education, as hypothesised.

**Table S6: *Post hoc* statistical power calculation at observed values for CSC (continuous score) x parental education (none/primary vs. higher)**

| **Interaction odds ratio (θ)**^a^ | **Power (%)** |
| --- | --- |
| 0.40 | 100 |
| 0.50 | 100 |
| 0.75 | 100 |
| 0.90 (as observed) | 82.1 |

^a^ Interaction odds ratios less than one indicated a stronger protective effect of CSC on case-control status in people with lower parental education, as hypothesised.
